# Comparison of electrode montages for ocular Vestibular Evoked Myogenic Potential (oVEMP) using air-conducted tone burst stimulation: Is there an optimal configuration?

**DOI:** 10.1016/j.bjorl.2026.101912

**Published:** 2026-09-22

**Authors:** Mônica Alcantara de Oliveira Santos, Karen de Carvalho Lopes, Patrícia Soares Montemagni, Caroline Martins dos Santos Leopoldo, Fernando Freitas Ganança

**Affiliations:** a Universidade Federal de São Paulo, Department of Otolaryngology and Head and Neck Surgery, São Paulo, SP, Brazil; b Santa Casa de Misericórdia de São Paulo, Department of Otolaryngology, São Paulo, SP, Brazil

**Keywords:** Ocular Vestibular Evoked Myogenic Potential (oVEMP), Electrode montage, Acoustic stimulation, Tone burst

## Abstract

**Objective:**

To compare three electrode montages – infraorbital (standard), chin and belly-tendon – for ocular Vestibular Evoked Myogenic Potential (oVEMP), using air-conducted 500 Hz tone burst stimulation in health adults.

**Methods:**

An observational cross-sectional study was conducted in 50 healthy volunteers (100 ears) aged 22‒55. oVEMP was recorded using three randomized electrode montages in all volunteers. N1 and P1 latencies, peak-to-peak amplitudes and asymmetry ratios were analyzed.

**Results:**

Belly-tendon montage was associated with shorter N1 and P1 latencies compared to both infraorbital and chin montages (p < 0.05) and no significant differences in peak-to-peak amplitudes or asymmetry ratios were observed. No ear side or sex differences were found, while a significant negative correlation between age and interamplitude was noted.

**Conclusions:**

While the belly-tendon montage demonstrated shorter N1 and P1 latencies, all three configurations provided comparable amplitude and asymmetry ratio values. These finds suggest that electrode montage choice may prioritize patient comfort, examiner experience and anatomical characteristics.

## Highlights

• Three oVEMP montages were compared: infraorbital, chin, belly-tendon.
• First study comparing three montages using air-conducted tone burst stimulation.
• Peak-to-peak amplitudes and asymmetry ratios were similar in all configurations.
• Belly-tendon montage had shorter latencies.
• Significant negative correlation was observed between age and interamplitude value.

## Introduction

Otolith organs ‒ saccule and utricle ‒ can be stimulated by loud sounds, eliciting muscular reflexes that can be recorded by surface electrodes. These muscular responses elicited by strong auditory stimuli are called Vestibular Evoked Myogenic Potential (VEMP).

VEMP was initially observed in cervical musculature, such as sternocleidomastoid muscle^1^ and, in 2005, Rosengren et al.^2^ described potentials recorded from surface electrodes placed under the eyes in response to acoustic stimulation, which they called ocular vestibular evoked myogenic potential, or oVEMP.

The oVEMP is a biphasic response with peaks around 10 and 15 ms, the first negative, called N1 or n10 and the second positive, P1 or p15, best recorded in the eye contralateral to the stimulated ear, which provides information regarding the function of the utricle and the superior vestibular nerve.^3^,^4^

Muscular responses, which mainly originate from the inferior oblique muscle, were initially better recorded when the electrodes were placed under the eyes with an upward gaze.^2^ Todd et al.^5^ proposed a montage with the active electrodes close to the eyes and the reference ones immediately below which has become the most widely used.^6^,^7^

Although this infraorbital position of the electrodes is the standard, some authors believe that it could contaminate the oVEMP recording^8^, ^9^, ^10^, ^11^, ^12^ because the reference electrode, due to its proximity to the muscle, would capture part of the muscle activity and this response captured on both electrodes (active and reference) could be understood as noise and, therefore, be canceled, leading to a reduction in potential amplitude. Based on this assumption, some studies placed the reference electrode further away from the active electrode.

Piker et al.^8^ suggested placing the reference electrode on the chin, showing an increase in amplitudes that ranged from 18%–43%. Zuniga et al.^10^ also observed an increase in amplitude in most cases, suggesting it could provide better noise cancellation; however, they observed greater variability in responses, with some ears demonstrating a decrease in amplitude and others an increase, suggesting more susceptibility to tension or relaxation of the surrounding muscles.

Sandhu et al.^9^ compared many positions of active and reference electrodes in order to evaluate optimal electrode positioning. Authors found that the highest amplitude responses were obtained with belly-tendon montage when the infraorbital active electrode was placed lateral to the midline, and the reference electrode was placed in the inner canthus of the eye. Makowiec at al.^11^ also used this montage and defended that reference contamination was lower because the reference electrodes were on electrically neutral tendons.

Mat et al.^12^ also found that belly-tendon electrode montage produced larger amplitudes than the chin and the infraorbital electrode montage, although they used narrow band CE-Chirps. Raveendran and Singh highlighted the importance of electrode montage in air-conducted oVEMP acquisition, demonstrating that montage selection influences not only waveform characteristics^13^ but also frequency-tuning properties,^14^ with the belly-tendon configuration yielding more favorable response patterns.

More recently, Scherer et al. evaluated a montage using the nasion as the reference site as an alternative to the standard configuration, showing that the discussion regarding electrode placement in oVEMP remains active and that new configurations are still being explored.^15^

The literature agrees that positioning the reference electrode further away from the active electrode provides better noise cancellation; however, it is difficult to compare the results, since studies use different patterns of stimulation (air or bone-conducted stimulus; sound level; click, tone burst or chirp). Moreover, it is important to determine which is the best position for acquiring responses, in order to capture the largest magnitude of the utriculo-ocular reflex and, furthermore, do not involve the contribution of unwanted surrounding muscles and even the capture of other noises that would harm the acquisition. This paper aims to evaluate the oVEMP response in healthy subjects and compare the three most studied electrode montages: standard (infraorbital), chin and belly-tendon; using the most commonly used stimulation: air-conducted 500 Hz tone burst.

## Methods

An observational cross-sectional study conducted at a tertiary hospital between April 2023 and February 2025, after approval by the Research Ethics Committee (64844422.8.1001.5505).

### Subjects

Volunteers of both sexes, aged between 18- and 60-years, were included if they had no prior history of otological or vestibular problems. Individuals with neurological or psychiatric diseases, or those suffering from an upper airway infection or with abnormal findings on otoscopy were excluded.

### oVEMP

The oVEMP measures were performed using a Chartr EP 200® auditory evoked potential device, with volunteers seated in a non-reclining chair, with sound stimulus by calibrated insert earphones (air conduction).

Air-conducted 500 Hz tone burst with an intensity of 97 dB HL, with a Blackman envelope (1 ms rise/fall time), stimulus rate of 5.1 s and a total of 200 stimulus repetitions were used. Furthermore, band-pass filtering was 1–1000 Hz.

Stimuli were delivered monoaurally, first in the right ear and then in the left ear and responses were recorded from the contralateral side. Subjects were instructed to maintain an upward gaze (30°) during the measure.

### oVEMP montages

Surface electrodes were positioned after cleaning the skin using abrasive paste Nuprep®, in order to keep impedance below 5 kΩ. The ground electrode was placed on the forehead Fpz position.

A random sequence previously determined (using the “RANDBETWEEN” function in Excel Office) of three different montages was carried out in each volunteer to avoid fatigue bias:

- Standard (infraorbital): Active – on the orbital margin under the center of the eye; Reference – 2 cm below the active electrode on the cheek.^5^
- Chin: Active – on the orbital margin under the center of the eye; Reference – on the chin (the reference inputs were jumpered).^8^^,^^10^
- Belly-tendon: Active – infraorbital lateral to eye midline; Reference – inner canthus of each eye.^9^^,^^11^

The potentials were replicated at least once to confirm repeatability. The average of two waves was taken using the Add function on the Chartr EP 200 device and used to mark N1 and P1, whose values were used for statistical analysis.

The variables analyzed were asymmetry ratios (%) (calculated as AR = (larger – smaller) / (larger + smaller) × 100), N1 and P1 absolute latencies (ms), and N1–P1 peak-to-peak amplitudes (μV). They were set by an otolaryngologist, with experience in electrophysiology, blinded to electrode positions. When potentials could not be recorded, the oVEMP was considered not recordable and marked as missing value.

### Statistical analysis

Quantitative values were compared in the standard, chin and belly-tendon positions. The Shapiro–Wilk test was used to test for normality. Means and standard deviations of the oVEMP latencies and peak-to-peak amplitudes were calculated for all electrode montages and compared using repeated measures ANOVA and t-tests for normally distributed data or the Friedman test and Wilcoxon signed-rank test for non-normal distributions.

The correlation between age and latencies or peak-to-peak amplitudes was evaluated using Spearman’s correlation coefficient.

Results from the right and left ears were compared using paired t-tests for the normally distributed data or Wilcoxon signed-rank tests for non-normal distributions. Statistical analysis was performed using SPSS v25. A p-value < 0.05 was considered significant and confidence level was 95%.

## Results

Fifty volunteers (100 ears), 25 males, 25 females, mean age 27.5-years (SD = 5.3; range 22–55), signed informed consent.

The results from the right and left ears were compared and showed no significant differences between sides, allowing pooling of data from both ears for analysis.

For the 100 ears tested, one volunteer exhibited no contralateral muscle responses in any montage. Three showed no response in the left ear and one in the right ear in all montages. Six individuals did not have responses only in the chin montage (1 left ear, 5 bilateral) and one did not have responses only in the belly-tendon montage (both ears).

These cases with absent oVEMP responses were treated as missing values and were not included in the analyses of latency and peak-to-peak amplitude for the corresponding montage. Therefore, the sample size varied across montages, with fewer analyzable recordings in the chin montage and, to a lesser extent, in the belly-tendon montage.

Asymmetry ratio showed no significant difference among electrode positions (Friedman test, p = 0.266).

An overall difference among montages was observed for N1 latency (repeated-measures ANOVA ‒ p = 0.005) and for P1 latency (Friedman test ‒ p < 0.001).

In pairwise comparisons, the belly-tendon montage was associated with shorter latencies than infraorbital montage for both N1 (9.94 ms, SD = 0.38 vs. 10.06 ms, SD = 0.45; paired *t*-test ‒ p = 0.029) and P1 (14.65 ms, SD = 0.84 vs. 15.14 ms, SD = 0.87; Wilcoxon signed-rank test ‒ p < 0.001).

Similarly, belly-tendon montage was associated with shorter latencies than chin montage for both N1 (9.94 ms, SD = 0.38 vs. 10.15 ms, SD = 0.52; paired *t*-test ‒ p = 0.002) and P1 (14.65 ms, SD = 0.84 vs. 15.20 ms, SD = 0.85; Wilcoxon signed-rank test ‒ p < 0.001).

There were no differences between N1 and P1 latencies obtained with infraorbital and chin montages (all p > 0.05).

No significant differences in peak-to-peak amplitude were found among electrode positions (Friedman test ‒ p = 0.791) (Fig. 1).

**Fig. 1:**
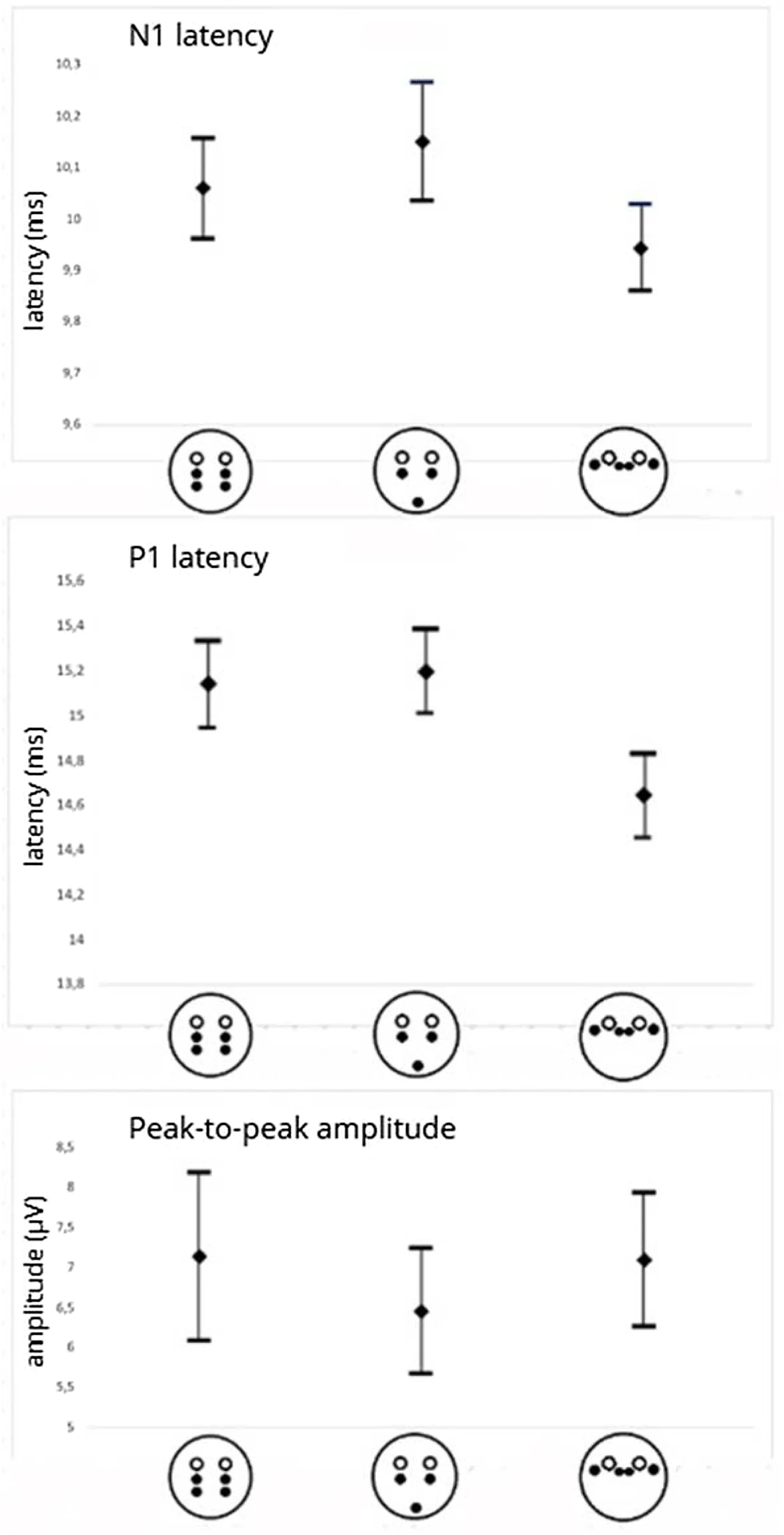
Mean values and confidence interval of N1 latency, P1 latency and peak-to-peak amplitude of oVEMP in healthy subjects, for three electrode montage protocols: = infraorbital;  = chin;  = belly-tendon.

Table 1 shows the mean values and standard deviations of N1 and P1 latencies, and peak-to-peak amplitudes.

**Table 1:** Mean values and standard deviations of N1 latency, P1 latency and peak-to-peak amplitude of oVEMP in healthy subjects, for three electrode montage protocols: infraorbital, chin and belly-tendon.

	Infraorbital		Chin		Belly-tendon		p
Variables	mean	SD	mean	SD	mean	SD	
N1 latency (ms)	10.06	0.45	10.15	0.52	9.94	0.38	0.005**
P1 latency (ms)	15.14	0.87	15.20	0.85	14.65	0.84	<0.001*
Peak-to-peak amplitude (μV)	7.14	4.74	6.46	3.55	7.10	3.79	0.791*

SD: standard deviation.

* Friedman.

** ANOVA.

For peak-to-peak amplitude no effect of the sex was observed (p = 0.056) but a significant negative correlation with age was revealed (*r*_s_ = −0.712, p = 0.005) indicating that as age increases, peak-to-peak amplitude values tend to decrease.

## Discussion

The scientific literature suggests that closer electrode placement during oVEMP testing may result in smaller potentials that are more difficult to identify and analyze. In this study, we investigated N1 and P1 latencies, as well as peak-to-peak amplitude, using three different electrode positions: infraorbital, chin, and belly-tendon. We aimed to evaluate whether electrode position would alter the response pattern. Our findings showed that the belly-tendon position was associated with shorter N1 and P1 latencies compared with infraorbital and chin positions. However, no significant differences in peak-to-peak amplitudes were observed among the electrode positions.

Shorter N1 latencies were first described by Makowiec et al.^11^ using the belly-tendon montage and in the same year, Leyssens et al.^16^ and Vanspauwen et al.^17^ also found significantly shorter P1 latencies when the reference electrode was placed on the medial upper part of the nose. Although Sandhu et al.^9^ did not find significant differences in mean latencies, there was a trend for the latency of N1 to become shorter as the reference electrode was placed further laterally. Based on these findings, which reflect anatomical and functional differences across regions, we believe that shorter latencies may be explained by the anatomical proximity of the electrodes to the tendinous belly of the muscle, which may facilitate activation and signal conduction, resulting in a faster response.

A balanced sample of men and women was selected because some authors, such as Shahnaz and David,^18^ have reported greater peak-to-peak amplitudes in men. However, in our sample, interamplitude values showed no relationship with sex. Regarding age, we included only individuals up to 55-years old, as it is well-documented that after the age of 60, many factors may contribute to absent potentials.^8^ In our study, cases with no response were not associated with older participants. However, we observed that interamplitude values tended to decrease with age, supporting the decision to exclude older individuals from the sample.

Additionally, there were no differences in the parameters analyzed between the right and left ears, suggesting that laterality does not influence the oVEMP response. This finding aligns with previous studies indicating that oVEMP responses in healthy individuals are generally symmetrical,^19^ allowing pooling the data from both ears for analysis.

Although most volunteers displayed consistent responses, a subset of participants did not exhibit responses, especially with the chin position. Six participants showed absent responses, either unilateral or bilateral, in this position. This difficulty may be attributed to greater interference from the capture of other facial muscle responses. These findings align with Zuniga et al.,^10^ who reported large variability in chin montage responses, suggesting that it was due to the contribution of surrounding muscles. Moreover, some participants had conditions that complicated electrode placement, such as the presence of beard hair at the chin position and small nasal anatomy (common in Asian subjects) at the belly-tendon position, which may have influenced signal acquisition despite ensuring impedance levels below 5 kΩ before starting the procedure.

Even though these physical conditions do not prevent oVEMP testing, they may introduce variability and, moreover, suggest that factors beyond stimulus efficiency or intensity should also be considered when selecting the best electrode position. It is often discussed in literature that a limiting factor in these studies is the loss of data from individuals who, despite being young and having no prior or current otologic history, fail to produce oVEMP recordings. Studies suggest that individual physiological variability, including anatomical differences and vestibular system sensitivity, may result in absent responses in up to 20% of cases, even in populations without evident disease.^6^^,^^20^ Piker et al.^21^ recommend adjusting the stimulus to 750 Hz or 1000 Hz when oVEMP responses are absent at 500 Hz. Similarly, Rosengren et al.^22^ suggest increasing stimulus intensity and instructing participants to maintain maximal up-gaze. However, in our study, due to its comparative nature across three electrode positions, we could not modify stimulus parameters.

Although latency differences were observed, no significant differences in peak-to-peak amplitudes were found among the electrode positions. As peak-to-peak amplitude is used to calculate asymmetry ratio, this suggests that electrode position did not impact the oVEMP outcome, and all of them can be used in vestibular evaluations. However, given the relatively large standard deviations observed for amplitude, small differences between montages cannot be excluded. Although our sample size was reasonably robust, larger studies may be required to clarify whether the lack of statistical significance reflects true equivalence or insufficient power to detect subtle effects.

From a clinical perspective, while the belly-tendon position exhibited shorter latencies, this difference is unlikely to significantly impact the speed of evaluation. Future studies may be needed to establish normative latency values specifically for this electrode position. The infraorbital position remains a valid technique, and our findings showed that all three positions yielded similar interamplitude values and asymmetry ratios. Thus, the choice of electrode position should depend on patient comfort (fewer electrodes on the face), physical aspects (such as beard) and anatomical characteristics (such as nose shape), and the examiner's experience. In our sample, the chin montage yielded absent responses more often, which may be relevant when selecting the recording setup in clinical practice.

Future studies should also examine different frequencies and stimulus types, since recent evidence suggests that the effects of electrode montage may go beyond conventional latency and amplitude comparisons^13^^,^^14^ and may enhance understanding of electrode positioning across various age groups.

## Conclusion

While the belly-tendon montage demonstrated shorter N1 and P1 latencies, all three configurations provided comparable amplitude and asymmetry ratio values using air-conducted 500 Hz tone burst stimulation in healthy adults.

## ORCID IDs

Patrícia Soares Montemagni: 0000-0001-6294-3504

Caroline Martins dos Santos Leopoldo: 0000-0003-2216-202X

### Declaration of Generative AI and AI-assisted technologies in the writing process

During the preparation of this manuscript, authors used ChatGPT 5.0 to improve language and spelling. After using this tool/service, the author reviewed and edited the content as needed and take full responsibility for the content of the publication.

## Funding

This research did not receive any specific grant from funding agencies in the public, commercial, or not-for-profit sectors.

## Data availability

The authors declare that all data are available in repository.

## Declaration of competing interest

The authors declare no conflicts of interest.
